# Prevalence and Molecular Characterization of Carbapenemase and Hypervirulence Genes in *Klebsiella pneumoniae* Isolates in Hospitals of Benghazi, Libya

**DOI:** 10.1155/ijm/2743383

**Published:** 2026-10-02

**Authors:** Sherif Asolimany, Raoudha Dziri, Ghada Ochi, Mohamed Iheb Ben Tekfa, Ghassan Tayh, Asma Elramli, Oumayma Kenza Chabbar, Hadda-Imene Ouzari, Naouel Klibi

**Affiliations:** ^1^ Department of Biology Laboratory of Microorganisms and Active Biomolecules Faculty of Sciences of Tunis Tunis El Manar University Tunis Tunisia utm.rnu.tn; ^2^ Department of Medical Laboratories Faculty of Public Health University of Benghazi Benghazi Libya uob.edu.ly; ^3^ Department of Microbiology and Immunology National School of Veterinary Medicine University of Manouba Sidi Thabet Tunisia uma.rnu.tn; ^4^ Department of Medical Technology College of Science and Technology Qaminis Libya cst.edu.bt

**Keywords:** carbapenemase genes, hypervirulence, *Klebsiella pneumoniae*, multidrug resistance, virulence genes

## Abstract

*Klebsiella pneumoniae* is a leading cause of hospital‐acquired infections, particularly in intensive care units (ICUs), where multidrug‐resistant (MDR) and hypervirulent strains represent major therapeutic challenges. This study investigated the phenotypic and molecular features of isolates recovered from ICU patients and the hospital environment in Benghazi, Libya. Antimicrobial susceptibility was determined using disk diffusion and the colistin broth disk elution method. Extended‐spectrum‐*β*‐lactamase (ESBL) and carbapenemase production were assessed phenotypically and confirmed by PCR and sequencing, whereas clonal relatedness was analyzed through OmpK36 typing. Hypervirulence was evaluated using the string test, PCR detection of virulence genes, and biofilm formation assays. A total of 56 isolates (46 clinical and 10 environmental) were included. All isolates were resistant to ceftriaxone, with high resistance rates to ceftazidime, cefotaxime, carbapenems, ciprofloxacin, and gentamicin. ESBL production was observed in 76% of clinical and 80% of environmental isolates, and 95% of all isolates were classified as MDR. A significant proportion of *K. pneumoniae* isolates (*n* = 15) exhibited resistance to colistin in the absence of detectable *mcr* genes. Strong and moderate biofilm production was detected in 30.4% and 17.9% of isolates, respectively. The *bla*_NDM-5_ was prevalent among clinical (45/46; 97.8%) and environmental (9/10; 90%) isolates. Similarly, *bla*_CTX-M-15_ was detected in 35/46 (76%) clinical isolates and 8/10 (80%) environmental isolates, whereas *bla*_OXA-48_ was detected in 26/46 (56.5%) clinical and 10/10 (100%) environmental isolates. A potentially hypervirulent phenotype was observed in 17.8% of the isolates, associated with the virulence genes *iuc*A, *iut*A, and *peg*‐344. OmpK36 Groups C and D predominated. These results highlight the alarming spread of MDR and hypervirulent strains in ICUs and underscore the need for reinforced infection control strategies.

## 1. Introduction

Hospital‐acquired infections emerged as a major public health concern following the widespread use of antibiotics. These infections contribute to increased healthcare costs, prolonged hospital stays, and elevated antibiotic consumption, which in turn leads to higher morbidity and mortality rates [1, 2]. Distinguishing between different types of infections often depends on the expression of pathogenicity‐related factors and the level of antimicrobial resistance exhibited by causative organisms. Multidrug‐resistant (MDR) pathogens represent a major global health threat, particularly members of the *Enterobacteriaceae* family that have developed resistance to carbapenem antibiotics [3]. *Klebsiella pneumoniae* is of particular concern due to its genetic plasticity, encompassing a diverse set of plasmid‐encoded and chromosomal genes. Genotypically, this species may exist as classic MDR strains or evolve into hypervirulent forms, each posing distinct challenges in clinical settings. The majority of *K. pneumoniae* infections have traditionally been attributed to classic strains (classical *Klebsiella pneumoniae* [cKp]), which are commonly associated with nosocomial environments and predominantly affect immunocompromised individuals. These cKp strains exhibit distinct characteristics that clearly differ from those of hypervirulent variants. The heightened virulence of hypervirulent *Klebsiella pneumoniae* (hvKp) is largely associated with genetic determinants located on large plasmids carrying multiple virulence‐associated genes [4].

Carbapenem resistance in *K. pneumoniae* is primarily driven by the production of carbapenemase enzymes, particularly those belonging to Ambler Classes A, B, and D. Additional resistance mechanisms include the coexpression of extended‐spectrum *β*‐lactamases (ESBLs) or AmpC *β*‐lactamases, often in combination with porin loss and/or increased activity of efflux pumps. The plasmid‐mediated nature of carbapenemase production is particularly concerning, as it facilitates efficient horizontal transfer of resistance genes. Consequently, this promotes the rapid spread of resistant strains within healthcare settings and increases the risk of dissemination into the broader community [5].

The concurrent presence of carbapenem‐hydrolyzing enzymes in cKp strains poses a significant challenge to current therapeutic options. This is attributable not only to their ability to confer resistance to nearly all *β*‐lactam antibiotics but also to their frequent association with genes mediating resistance to non‐*β*‐lactam agents [6]. The increasing prevalence of MDR *K. pneumoniae* in hospital‐acquired infections has therefore markedly limited effective therapeutic strategies. *β*‐lactamases such as NDM‐1, OXA‐48, OXA‐181, KPC, and cefotaxime (CTX)‐M‐15 have emerged as major contributors, conferring resistance to a wide range of antibiotics, including penicillins, carbapenems, cephalosporins, and sulfamethoxazole [7].

hvKp clones are frequently characterized by the presence of mobile virulence plasmids that carry accessory genes responsible for enhanced pathogenicity [8, 9]. These plasmids typically encode genes involved in aerobactin siderophore biosynthesis (*iuc*A), regulation of the hypermucoid phenotype (*rmp*A), and a putative metabolite transporter (*peg*‐344). These three genetic markers (*iuc*A, *rmp*A, and *peg-*344) are commonly colocated on large virulence‐associated plasmids, although structural variability among plasmids has been reported [9, 10]. In clinical microbiology, the detection of these genotypic markers has become a practical and reliable approach for the rapid identification of hypervirulent strains, offering a more consistent alternative to phenotype‐based methods [11, 12].

A study conducted in Libya reported a high burden of antimicrobial resistance in *K. pneumoniae*, with MDR and extensively drug‐resistant (XDR) strains accounting for 89% and 56% of the isolates, respectively, highlighting a significant public health concern [13]. In recent years, the impact of MDR Gram‐negative bacteria has increased markedly in Libya, particularly in intensive care units (ICUs), where *K. pneumoniae* has exhibited the highest resistance rates to commonly used antibiotics among ICU patients at Benghazi Medical Center [14]. Owing to the scarcity of data on carbapenemase genes in Libya, information on hypervirulence‐associated genes in *K. pneumoniae* is entirely lacking both in Libya and across North Africa. Therefore, the present study is aimed at detecting carbapenemase and hypervirulence‐associated genes, evaluating antimicrobial resistance profiles, and characterizing phenotypic patterns and their relationships with corresponding genotypes among *K. pneumoniae* isolates recovered from ICUs in hospitals in Benghazi, Libya.

## 2. Materials and Methods

### 2.1. Bacterial Isolation

Between November 2022 and April 2023, a total of 46 samples were randomly collected from patients admitted to the ICU in Benghazi. The samples included wound (*n* = 15), blood (*n* = 8), urine (*n* = 8), burn swabs (*n* = 6), pus (*n* = 3), cerebrospinal fluid (*n* = 2), sputum (*n* = 2), and suction bottle fluid (*n* = 2). Additionally, 10 environmental samples were obtained from various surfaces, including the central venous line (*n* = 4), catheter (*n* = 2), endotracheal tube (*n* = 2), and trolley (*n* = 2) within the ICU. The inclusion criteria are as follows: (i) clinical samples from patients admitted to the ICU, (ii) environmental samples from the ICU, and (iii) nonrepeated samples. The exclusion criteria included duplicate samples and contaminated samples.

All collected specimens were promptly transported to the microbiology laboratory for processing. Each sample was inoculated onto MacConkey agar (MCA, Merck, Germany) and incubated at 37°C for 24 h. Following incubation, Gram staining was performed for preliminary classification of the isolates. Bacterial isolation was performed on MacConkey agar (bioMérieux, Marcy‐l’Etoile, France), followed by identification using the API 20E system (bioMérieux, Marcy‐l’Etoile, France). All isolates were subsequently confirmed by polymerase chain reaction (PCR) and sequencing of 16S DNA using forward and reverse primers (F: 5 ^′^‐AGAGTTTGATGGCTCAG‐3 ^′^/R: 5 ^′^‐TACGGCTACCTTGTTACGACTT‐3 ^′^) as previously described [15]. Thermal cycling reactions consisted of an initial denaturation at 94°C for 3 min, followed by 35 cycles of denaturation at 94°C for 45 s, annealing at 55°C for 1 min, and extension at 72°C for 2 min, followed by a single final extension at 72°C for 6 min.

Isolates were frozen at −30°C in brain–heart infusion (BHI) broth with 15% glycerol until processed for further experimentation.

### 2.2. Antimicrobial Susceptibility Testing

Antibiotic susceptibility pattern of *K. pneumoniae* isolates was determined using the Kirby–Bauer disk diffusion method on Mueller–Hinton agar (Merck), following the guidelines of the Clinical and Laboratory Standards Institute (CLSI) [16]. The antibiotic disks (Bio‐Rad, Marnes‐la‐Coquette, France) tested included the following (micrograms per disk): amoxicillin–clavulanic acid (AMC) (20/10), piperacillin–tazobactam (30/6), ceftriaxone (30), ceftazidime (CAZ) (30), CTX (30), aztreonam (30), cefoxitin (30), cefuroxime (30), imipenem (10), meropenem (10), gentamicin (10), amikacin (30), ciprofloxacin (5), levofloxacin (5), trimethoprim–sulfamethoxazole (1.25/23.75), and tigecycline (15) (Oxoid Basingstoke, United Kingdom). All inoculated plates were incubated aerobically at 37°C for 18–24 h. The results were interpreted according to the CLSI (2022) recommendations. Colistin susceptibility testing of *K. pneumoniae* isolates was performed using the colistin broth disk elution (CBDE) method, as recently described by Alhamwi and Öksüz [16]. Isolates were subcultured on BHI agar to ensure purity and viability. A bacterial suspension equivalent to a 0.5 McFarland was prepared in sterile physiological saline and inoculated into test tubes containing 10 mL of cation‐adjusted Mueller–Hinton broth. Colistin disks were added at concentrations of 10, 20, and 40 *μ*g, with a colistin‐free tube included as a growth control. Following mixing, the tubes were incubated at 35°C–37°C for 16–20 h, after which bacterial growth was assessed visually. The presence or absence of visible growth was used to determine colistin resistance or susceptibility [17].

### 2.3. Detection of ESBL and Metallo‐*β*‐Lactamase (MBL) Production

ESBL production was determined using the combined disk method. This assay employed AMC (30 *μ*g), CTX (30 *μ*g), and CAZ (30 *μ*g) disks. Isolates were considered ESBL producers when an increase of ≥ 5 mm in the inhibition zone diameter was observed for the clavulanic acid–containing disk compared to the corresponding antibiotic disk without clavulanic acid. MBL production was assessed using the combined disk test (CDT) with ethylenediaminetetraacetic acid (EDTA). A bacterial suspension equivalent to 0.5 McFarland turbidity was prepared from an overnight culture and uniformly inoculated onto Mueller–Hinton agar plates. Two imipenem disks (IPM, 10 *μ*g) were placed on the agar surface, and 10 *μ*L of the 0.5 M EDTA solution was added to one of the disks. The plates were incubated at 35°C for 18–24 h. An increase in inhibition zone diameter of ≥ 7 mm around the imipenem‐EDTA disk compared with the imipenem‐only disk was interpreted as indicative of MBL production [15].

### 2.4. Detection of Antimicrobial Resistance Genes

The bacterial genomic DNA was extracted using thermal lysis (boiling lysis). PCR and sequencing were employed to detect antimicrobial resistance genes, including *bla*_NDM_, *bla*_KPC_, *bla*_VIM_, *bla*_GES_, *bla*_OXA-48_, and *bla*_SHV_. PCR amplification was performed using primers and conditions previously described [18, 19]. Colistin‐resistant *K. pneumoniae* isolates were screened for the presence of *mcr*‐1 to *mcr*‐9 genes using two multiplex PCR assays with primers published elsewhere [20, 21]. The PCR products were separated by electrophoresis on a 2% agarose gel stained with ethidium bromide and run at 100 V for 35 min. Positive control strains from the Laboratory of Microorganisms and Active Biomolecules were included for each target gene.

### 2.5. Characterization of hvKp Isolates

#### 2.5.1. Mucoid Phenotype Determination

The mucoid phenotype associated with the hypermucoviscosity of *K. pneumoniae* was determined using the string test, as previously described [22]. A positive result was defined by the formation of a viscous string exceeding 5 mm in length when a bacterial colony was stretched using an inoculation loop.

#### 2.5.2. Biofilm Formation Assay

Biofilm formation was performed using a 96‐well microtiter plate assay following the guidelines of Sabença et al. [23]. Bacterial suspensions were adjusted to 0.5 McFarland (1.5 × 10^8^ CFU/mL), and 100 *μ*L was added per well. *Pseudomonas aeruginosa* ATCC 27853 and sterile TSB served as positive and negative controls, respectively. After incubation at 37°C for 24 h, wells were washed, methanol‐fixed (15 min), stained with 1% crystal violet (15 min), and the bound dye was solubilized with 30% acetic acid. Biofilm biomass was quantified by measuring the optical density (OD) at 630 nm (Multiskan GO, Thermo Scientific). The optical density cut‐off (ODc) was defined as the mean OD of the negative control + (3 × standard deviation), and isolates were classified as non (OD ≤ ODc), weak (ODc < OD ≤ 2), moderate (2 × ODc < OD ≤ 4 × ODc), or strong biofilm producers (OD > 4 × ODc) according to ODc‐based criteria.

#### 2.5.3. Molecular Identification of hvKp

All *K. pneumoniae* strains were screened for the presence of hypervirulence‐associated genes using PCR. The following genes were targeted: *iuc*A, *iut*A, *peg*1‐344, *peg*2‐344, *rmp*A, *iro*B, *ybt*, *kfu*, *allS*, *mag*A, *fim*H, *mrk*D, *Wab*G, and *uge* (Table 1).

**Table 1:** Primers, product sizes, and annealing temperatures used for virulence gene detection in this study.

Primer name	Primer sequence (5 ^′^→3 ^′^)	Amplicon size (bp)	Annealing temperature (°C)	References
*peg1-344*	F: GCGGGAAAGGACAGAAAGCCAGTG R: GAGGGAAGATGAGAAATACGAGC	508	56	[24]
*peg2-344*	F: GCGGGAAAGGACAGAAAGCCAGTG R: GAGGGAAGATGAGAAATACGAGC	332	56	[24]
*iucA*	F: AATCAATGGCTATTCCCGCTG R: CGCTTCACTTCTTTCACTGACAGG	239	62	[24]
*iutA*	F: GCCGCTAGGTTGGTGATGT R: CTCTGGTCGTGCTGGTTGA	300	61	[24]
*magA*	F: GGTGCTCTTTACATCATTGC R: GCAATGGCCATTTGCGTTAG	1282	51	[24]
*rmpA*	F: GAGTATTGGTTGACAGCAGGAT R: AGCCGTGGATAATGGTTTACAA	516	53	[24]
*kfu*	F: ATAGTAGGCGAGCACCGAGA R: AGAACCTTCCTCGCTGAACA	520	60	[24]
*allS*	F: CCGAAACATTACGCACCTTT R: ATCACGAAGAGCCAGGTCAC	508	60	[24]
*ybt*	F: GACGGAAACAGCACGGTAAA R: GAGCATAATAAGGCGAAAGA	242	60	[24]
*iroB*	F: GTGTTGGATTCCGCCAGTGA R: TTCCGCCGCTACCTCTTCA	366	61	[24]
*uge*	F: TCTTCACGCCTTCCTTCACT R: GATCATCCGGTCTCCCTGTA	534	53	[24]
*Fim-H*	F: TGCTGCTGGGCTGGTCGATG R: GGGAGGGTGACGGTGACATC	688	62	[24]
*WabG*	F: ACCATCGGCCATTTGATAGA R: CGGACTGGCAGATCCATATC	683	53	[24]
*mrkD*	F: TTCTGCACAGCGGTCCC R: GATACCCGGCGTTTTCGTTAC	240	53	[24]
*OmpK36 Group A*	F: GAAGGCGCTCTGTCTCCTA R: TGCCATCATAGATGTCATAGG	97	60	[24]
*OmpK36 Group B*	F: CGGTCGTGGCGCGCAGAAA R: GGTTGTTCTGA TCGTCGGTA	125	60	[24]
*OmpK36 Group C*	F: CAACAACGGTCGTGGTTGGA R: CCCAGTGCCGGAACACTATT	144	60	[24]
*OmpK36 Group D*	F: GAAGGTACTTCTCCGACCAA R: AATCAGATTCTCCGTTGCCG	283	60	[24]

#### 2.5.4. Determination of Clonal Relatedness Using OmpK36 Typing

The clonal relatedness among hvKp isolates was assessed using OmpK36 typing. This analysis was performed by PCR as described by Yan et al. [25] (Table 1).

## 3. Results

### 3.1. Characteristics and Sources of *K. pneumoniae* Isolates

A total of 56 K*. pneumoniae* isolates were included in this study. Among them, 46 clinical isolates were collected from patients admitted to ICUs at two hospitals in Benghazi: Al‐Jalaa Hospital (A) and Benghazi Medical Center (B) (Table 2). Most isolates were obtained from wound specimens (15/46; 32.6%), followed by blood (8/46; 17.4%), urine (8/46; 17.4%), burn specimens (6/46; 13%), pus (3/46; 6.5%), sputum (2/46; 4.3%), cerebrospinal fluid (2/46; 4.3%), and suction bottle samples (2/46; 4.3%). In addition, 10 environmental isolates were recovered from ICU surfaces, including central venous lines (4/10; 40%), catheters (2/10; 20%), endotracheal tubes (2/10; 20%), and a trolley (2/10; 20%) (Table 3).

**Table 2:** Phenotypic and molecular characteristics of ICU clinical *K. pneumoniae* isolates.

Hospital	Source	Resistance antibiotic	ESBL test	EDTA test	String test	MIC colistin (*μ*g/mL)	Resistance genes	Virulence factor genes	OmpK36 typing
A	Wound	CRO, AZM, CIP, CN, CAZ, TZP, LEV, CTX, AK, MEM, IMP, AMC, and COL	Negative	Positive	Positive	> 4	*OXA*‐48 and *NDM*‐5	*Uge*, *peg1-344*, *rmpA*, *WabG*, *iutA*, *ybt*, *iroB*, and *mrkD*	C
A	Urine	CRO, AZM, CIP, CN, CAZ, TZP, CTX, LEV, AK, MEM, IMP, and AMC	Positive	Positive	Negative	≤ 1	*OXA-*48, *NDM*‐5, *CTXM-*15, and *SHV*‐11	*Uge*, *peg1-344*, *rmpA*, *WabG*, *iutA*, *ybt*, *iroB*, and *mrkD*	C
A	Wound	CRO, CIP, CAZ, TZP, CTX, LEV, AK, MEM, IMP, and AMC	Positive	Positive	Negative	≤ 1	*OXA-*48, *NDM*‐5, and *CTXM-*15	*Uge*, *rmpA*, *ybt*, and *iroB*	NT
A	Blood	CRO, AZM, CN, CAZ, TZP, CTX, and IMP	Positive	Positive	Positive	≤ 1	*OXA-*48, *NDM*‐5, and *CTXM*‐15	*Uge*, *rmpA*, *iuc*A, *magA*, *Wab*, *ybt*, *iroB*, and *mrkD*	D
A	Wound	CRO, CN, CAZ, TZP, CTX, AK, MEM, IMP, and COL	Positive	Positive	Positive	4	*NDM*‐5, *CTXM-*15, and *SHV-*11	*Uge*, *peg-344*, *rmpA*, *iuc*A, *WabG*, *iutA*, *ybt*, and *iroB*	D
A	Wound	CRO, AZM, CIP, CN, CAZ, TZP, CTX, LEV, MEM, IMP, AMC, and COL	Positive	Positive	Negative	> 4	*NDM-*5, *CTXM-*15, and *SHV*‐31	*Uge*, *peg1-344*, *rmpA*, *iuc*A, *WabG*, *iutA*, *ybt*, *iroB*, *allS*, and *mrkD*	B
A	Wound	CRO, CIP, CN, CAZ, TZP, CTX, LEV, AK, MEM, IMP, and AMC	Positive	Positive	Negative	≤ 1	*OXA-*48, *NDM-*5, and *CTXM-*15	*Uge*, *peg1-344*, *rmpA*, *iuc*A, *WabG*, *iutA*, *ybt*, and *iroB*	NT
A	Burn	CN, CAZ, TZP, CTX, LEV, AK, MEM, IMP, and CRO	Positive	Positive	Positive	≤ 1	*NDM*‐5, *CTXM*‐15, and *SHV*‐11	*Uge*, *rmpA*, *iuc*A, *magA*, *WabG*, *iutA*, *ybt*, and *iroB*	D
A	Burn	CRO, CIP, CN, CAZ, TZP, CTX, AK, MEM, and COL	Positive	Positive	Positive	> 4	*OXA-*48, *NDM-*5, *CTXM-*15, and *SHV-*11	*Uge*, *rmpA*, *iuc*A, WabG, *iutA*, *ybt*, and *iroB*	D
A	Urine	CRO, CN, CAZ, TZP, CTX, LEV, AK, AMC, MEM, and IMP	Positive	Positive	Negative	≤ 1	*OXA-*48, *NDM-*5, and *CTXM-*15	*Uge*, *iuc*A, *WabG*, *iutA*, *ybt*, and *iroB*	D
A	Wound	CRO, CN, CAZ, TZP, CTX, LEV, AK, MEM, IMP, and AMC	Positive	Positive	Positive	2	*OXA-*48, *NDM-*5, and *CTXM-*15	*Uge*, *rmpA*, *iuc*A, *WabG*, *iutA*, *ybt*, *allS*, *iroB*, and *mrkD*	C
A	CSF	CRO, AZM, CIP, CN, CAZ, TZP, LEV, AK, MEM, and IMP	Positive	Positive	Positive	≤ 1	*OXA-*48, *NDM-*5, and *CTXM-*15	*Uge*, *peg1-344*, *rmpA*, *iuc*A, *WabG*, *kfu*, *ybt*, and *iroB*	NT
A	Burn	CRO, AZM, CIP, CN, CAZ, TZP, LEV, MEM, IMP, and SXT	Positive	Positive	Negative	≤ 1	*OXA-*48, *NDM-*5, and *CTXM*‐15	*Uge*, *peg-344*, *WabG*, *ybt*, and *iroB*	NT
A	Sputum	CRO, CIP, CN, CAZ, TZP, CTX, LEV, AK, MEM, IMP, AMC, and COL	Negative	Positive	Positive	4	*OXA-*48, *NDM*‐5, *CTXM-*15, and *SHV-12*	*Uge*, *peg1-344*, *rmpA*, *magA*, *iuc*A, *WabG*, *kfu*, *ybt*, and *iroB*	NT
A	Burn	CRO, CIP, CN, CAZ, TZP, CTX, LEV, AK, and MEM	Negative	Positive	Positive	≤ 1	*NDM-*5 and *CTXM-*15	*Uge*, *rmpA*, *magA*, *iuc*A, *WabG*, *iutA*, *ybt*, *iroB*, and *mrkD*	C
A	Burn	CRO, CN, CAZ, TZP, CTX, LEV, AK, MEM, and IMP	Positive	Positive	Positive	≤ 1	*OXA*‐48 and *NDM-*5	*Uge*, *rmpA*, *icuA*, *WabG*, *iutA*, *ybt*, *iroB*, and *mrkD*	B
A	Blood	CRO, CIP, CAZ, TZP, CTX, LEV, MEM, IMP, and COL	Positive	Positive	Positive	4	*NDM-*1	*Uge*, *magA*, *WabG*, *iutA*, *kfu*, *ybt*, *iroB*, and *mrkD*	B
A	Blood	CRO, CIP, CN, CAZ, CTX, LEV, MEM, and IMP	Positive	Positive	Negative	≤ 1	*NDM-*5	*Uge*, *WabG*, *ybt*, and *iroB*	D
A	Blood	CRO, CIP, CN, CAZ, TZP, CTX, LEV, MEM, and IMP	Negative	Positive	Positive	2	*NDM-*5 and *CTXM*‐15	*Uge*, *rmpA*, *magA*, *iuc*A, *WabG*, *iutA*, *kfu*, *ybt*, and *iroB*	D
A	Wound	CRO, AZM, CIP, CN, CAZ, TZP, CTX, MEM, IMP, SXT, and COL	Positive	Positive	Positive	> 4	*OXA-*48, *NDM-*5, and *CTXM-*15	*Uge*, *peg1-344*, *rmpA*, *mAg*, *iuc*A, *WabG*, *iutA*, *ybt*, and *iroB*	C
A	Urine	CRO, CIP, CN, CAZ, TZP, CTX, LEV, AK, MEM, IMP, and COL	Positive	Positive	Positive	4	*OXA-*48, *NDM-5*, and *SHV-*11	*Uge*, *rmpA*, *magA*, *iuc*A, *WabG*, *iutA*, *ybt*, *iroB*, and *mrkD*	C
A	Wound	CRO, CIP, CN, CAZ, TZP, CTX, LEV, MEM, and AK	Positive	Positive	Positive	≤ 1	*OXA-*48, *NDM-5*, and *CTXM-*15	*Uge* and *mag*	D
A	Suction bottle	CRO, CIP, CN, CAZ, TZP, CTX, LEV, MEM, and IMP	Positive	Positive	Positive	≤ 1	*NDM-*5 and *CTXM-*15	*Uge*, *magA*, *iuc*A, *WabG*, *iutA*, *ybt*, *iroB*, and *mrkD*	D
A	CSF	CRO, CIP, CN, CAZ, TZP, CTX, LEV, AK, MEM, IMP, and COL	Positive	Positive	Positive	4	*NDM*‐5, *CTXM-*15, and *SHV-*11	*Uge*, *magA*, *iuc*A, *WabG*, *iutA*, *ybt*, *iroB*, and *mrkD*	B
A	Wound	CRO, CIP, CN, CAZ, TZP, CTX, LEV, AK, and IMP	Negative	Positive	Positive	≤ 1	*NDM-*5	*Uge*, *peg1-344*, *rmpA*, *magA*, *iuc*A, *iutA*, *WabG*, *kfu*, *ybt*, and *iroB*	B
A	Pus	CRO, CIP, CN, CAZ, TZP, CTX, LEV, AK, MEM, and IMP	Positive	Positive	Negative	≤ 1	*OXA-*48 and *NDM-*5	*Uge*, *WabG*, *kfu*, *ybt*, and *iroB*	NT
B	Blood	CRO, AZM, CIP, CN, CAZ, TZP, CTX, LEV, AK, SXT, MEM, IMP, TIG, AMC, and COL	Positive	Positive	Positive	4	*OXA-*48, *NDM-*5, *CTXM-*15, and *SHV*144	*Uge*, *peg1-344*, *rmpA*, *iuc*A, *iutA*, *WabG*, *kfu*, *ybt*, and *iroB*	B
B	Sputum	CRO, CIP, CN, CAZ, TZP, CTX, LEV, AK, MEM, and IMP	Positive	Positive	Positive	≤ 1	*NDM-*5 and *CTXM-*15	*Uge*, *rmpA*, *WabG*, *kfu*, *ybt*, and *iroB*	NT
B	Urine	CRO, CIP, CN, CAZ, TZP, CTX, LEV, AK, MEM, and IMP	Negative	Positive	Negative	≤ 1	*NDM*‐5	*Uge*, *iuc*A, *WabG*, *ybt*, and *iroB*	C
B	Blood	CRO, AZM, CIP, CN, CAZ, TZP, CTX, LEV, AK, SXT, and MEM	Negative	Positive	Positive	≤ 1	*OXA-*48 and *NDM-*5	*Uge*, *rmpA*, *iuc*A, *iutA*, *WabG*, *kfu*, *allS*, and *iroB*	NT
B	Wound	CRO, AZM, CIP, CN, CAZ, TZP, CTX, LEV, AK, SXT, MEM, and IMP	Positive	Positive	Negative	≤ 1	*NDM-*5 and *CTXM-*15	*Uge*, *iuc*A, *WabG*, *kfu*, *ybt*, and *iroB*	D
B	Wound	CRO, AZM, CIP, CN, CAZ, TZP, CTX, LEV, AK, SXT, MEM, and IMP	Positive	Positive	Positive	2	*OXA-*48 and *NDM-5*	*Uge*, *rmpA*, *iuc*A, *magA*, *iutA*, *WabG*, *ybt*, *iroB*, and *mrkD*	C
B	Urine	CRO, AZM, CIP, CN, CAZ, TZP, CTX, LEV, AK, SXT, and MEM	Positive	Positive	Positive	≤ 1	*NMD-*5 and *CTXM-*15	*Uge*, *peg1-344*, *rmpA*, *iutA*, *WabG*, *kfu*, *ybt*, and *iroB*	B
B	Burn	CRO, AZM, CIP, CN, CAZ, TZP, CTX, LEV, AK, STX, MEM, IMP, and COL	Positive	Positive	Negative	4	*OXA*‐48 and *NDM*‐5	*Uge*, *peg1-344*, *iuc*A, *WabG*, *ybt*, *iroB*, and *mrkD*	C
B	Urine	CRO, CIP, CN, CAZ, TZP, CTX, LEV, AMK, MEM, and IMP	Positive	Positive	Negative	≤ 1	*NDM-*5, *CTXM-*15, and SHV*-*11	*Uge*, *iuc*A, *WabG*, *kfu*, *ybt*, and *iroB*	C
B	Urine	CRO, CIP, CN, CAZ, TZP, CTX, LEV, AK, MEM, and IMP	Positive	Positive	Positive	≤ 1	*NDM-*5 and *CTXM-*15	*Uge*, *rmpA*, *iuc*A, *WabG*, *kfu*, *ybt*, and *iroB*	C
B	Blood	CRO, AZM, CIP, CN, CAZ, TZP, CTX, LEV, AK, MEM, IMP, SXT, TIG, and COL	Negative	Positive	Positive	4	*OXA-*48, *NDM-*5, *CTXM-*15, and *SHV*11	*Uge*, *rmpA*, *peg1-344*, *iutA*, *WabG*, *ybt*, *iroB*, and *mrkD*	D
B	Wound	CRO, AZM, CIP, CN, CAZ, TZP, CTX, LEV, AK, MEM, IMP, and COL	Positive	Positive	Positive	4	*OXA*‐48 and *NDM-*5	*Uge*, *peg1-344*, *rmpA*, *iuc*A, *magA*, *iutA*, *WabG*, *ybt*, and *iroB*	B
B	Blood	CRO, AZM, CIP, CN, CAZ, TZP, CTX, LEV, AK, MEM, and IMP	Positive	Positive	Positive	≤ 1	*NDM-*5 and *CTXM-*15	*Uge*, *rmpA*, *iutA*, *WabG*, *ybt*, *iroB*, and *mrkD*	C
B	Pus	CRO, AZM, CIP, CN, CAZ, TZP, CTX, LEV, AK, MEM, and IMP	Positive	Positive	Positive	≤ 1	*NDM*‐5	*Uge*, *peg1-344*, *rmpA*, *magA*, *WabG*, *iutA*, *ybt*, *iroB*, and *mrkD*	C
B	Suction bottle	CRO, CIP, CN, CAZ, TZP, CTX, LEV, AK, MEM, and IMP	Negative	Positive	Positive	≤ 1	*NDM-*5	*Uge*, *magA*, *WabG*, *iutA*, *ybt*, *iroB*, and *mrkD*	D
B	Wound	CRO, CIP, CN, CAZ, TZP, CTX, LEV, AK, MEM, IMP, SXT, and TIG	Positive	Positive	Positive	≤ 1	*NDM-5* and *CTXM-*15	*Uge*, *rmpA*, *iuc*A, *iutA*, *WabG*, *kfu*, *ybt*, and *iroB*	C
B	Wound	CRO, CIP, CN, CAZ, TZP, CTX, LEV, AK, MEM, and IMP	Positive	Positive	Negative	≤ 1	*OXA-*48, *NDM*‐5, and *CTXM-*15	*Uge*	B
B	Wound	CRO, CIP, CN, CAZ, TZP, CTX, LEV, MEM, IMP, SXT, TIG, and AK	Positive	Positive	Negative	≤ 1	*OXA-*48 and *NDM-*5	*Uge*, *iuc*A, *WabG*, *ybt*, *iroB*, and *allS*	NT
B	Urine	CRO, CIP, CN, CAZ, CTX, LEV, AK, and IMP	Negative	Positive	Negative	2	*OXA-*48 and *NDM-*5	*Uge*	C
B	Pus	CRO, AZM, CIP, CN, CAZ, TZP, CTX, LEV, AK, MEM, IMP, AMC, and COL	Negative	Positive	Positive	4	*OXA*‐48, *NDM-*5, *CTXM-*15, and *SHV-*11	*Uge*, *peg1-344*, *iuc*A, *rmpA*, *magA*, *WabG*, *iutA*, *ybt*, *iroB*, and *allS*	D

Abbreviations: AK, amikacin; AMC, amoxicillin–clavulanic acid; AZM, azithromycin; CAZ, ceftazidime; CIP, ciprofloxacin; CN, gentamicin; COL, colistin; CRO, ceftriaxone; CSF, cerebrospinal fluid; CTX, cefotaxime; ICU, intensive care unit; IMP, imipenem; LEV, levofloxacin; MEM, meropenem; NT, no typable; SXT, trimethoprim–sulfamethoxazole; TIG, tigecycline; TZP, piperacillin–tazobactam.

**Table 3:** Phenotypic and molecular characteristics of *K. pneumoniae* isolates collected from the ICU environment.

Hospital	Source	Resistance antibiotic	ESBL test	EDTA test	String test	Colistin MIC (*μ*g/mL)	Resistance genes	Virulence factor genes	OmpK36 typing
A	CVL	CRO, AZM, CIP, CTX, CAZ, CN, LEV, AK, MEM, and FOX	Positive	Positive	Negative	≤ 1	*OXA*‐48, *NDM*‐*5*, and *CTXM-*15	*Uge*, *rmpA*, *WabG*, *ybt*, and *iroB*	B
A	Catheter	CRO, AZM, CIP, TZP, CTX, LEV, MEM, AK, SXT, IMP, and TIG	Positive	Positive	Negative	2	*OXA-*48, *NDM-*5, and *CTXM-*15	*Uge*, *rmpA*, *iutA*, *WabG*, *ybt*, *iroB*, and *allS*	C
A	CVL	CRO, AZM, CIP, TZP, CTX, CAZ, CN, LEV, MEM, AK, SXT, IMP, and TIG	Positive	Positive	Positive	≤ 1	*OXA-*48, *NDM-*5, and *CTXM-*15	*Uge*, *peg1-344*, *iucA*, *rmpA*, *WabG*, *iutA*, *ybt*, and *iroB*	C
A	Trolley	CRO, AZM, CIP, TZP, CTX, CAZ, CN, LEV, MEM, AK, SXT, IMP, and TIG	Negative	Positive	Positive	≤ 1	*OXA-*48 and *NDM-*5	*Uge*, *peg1-344*, *iucA*, *rmpA*, *WabG*, *ybt*, and *iroB*	C
A	CVL	CRO, AZM, CIP, TZP, CTX, CAZ, LEV, MER, SXT, TIG, IMP, and FOX	Positive	Positive	Positive	≤ 1	*OXA-*48, *NDM-*5, and *CTXM-*15	*Uge*, *iutA*, *rmpA*, *WabG*, *ybt*, *iroB*, and *mrkD*	B
B	ETT	CRO, AZM, CIP, TZP, CTX, CAZ, CN, LEV, MEM, AK, SXT, IMP, and TIG	Negative	Positive	Negative	≤ 1	*OXA-*48 and *NDM-*5	*Uge*, *iutA*, *WabG*, *ybt*, *iroB*, and *mrkD*	D
B	CVL	CRO, AZM, CIP, TZP, CTX, CAZ, CN, LEV, MEM, AK, SXT, IMP, and TIG	Positive	Positive	Positive	≤ 1	*OXA-*48, *NDM-*5, *CTXM-*15, and *SHV-*11	*Uge*, *iuc*A, *magA*, *WabG*, *ybt*, *iroB*, and *mrkD*	B
B	Trolley	CRO, AZM, CIP, TZP, CTX, CAZ, CN, LEV, MEM, AK, SXT, IMP, and TIG	Positive	Positive	Positive	≤ 1	*OXA-*48, *NDM-*5, and *CTXM*‐15	*Uge*, *rmpA*, *magA*, *WabG*, *kfu*, *ybt*, *iroB*, and *mrkD*	C
B	Catheter	CRO, AZM, CIP, TZP, CTX, CAZ, CN, LEV, MEM, AK, SXT, IMP, and COL	Positive	Positive	Positive	> 4	*OXA-*48, *NDM-*5, and *CTXM-*15	*Uge*, *peg1-344*, *iucA*, *rmpA*, *WabG*, *iutA*, *ybt*, *iroB*, and *mrkD*	C
B	ETT	CRO, AZM, CIP, TZP, CTX, CAZ, CN, LEV, MEM, AK, SXT, IMP, and TIG	Positive	Negative	Negative	≤ 1	*OXA-*48 and *CTXM*‐15	*Uge*, *iutA*, *WabG*, *kfu*, *ybt*, and *iroB*	C

Abbreviations: AK, amikacin; AMC, amoxicillin–clavulanic acid; AZM, azithromycin; CAZ, ceftazidime; CIP, ciprofloxacin; CN, gentamicin; COL, colistin; CRO, ceftriaxone; CTX, cefotaxime; CVL, central venous line; ETT, endotracheal tube; FOX, cefoxitin; ICU, intensive care unit; IMP, imipenem; LEV, levofloxacin; MEM, meropenem; SXT, trimethoprim–sulfamethoxazole; TIG, tigecycline; TZP, piperacillin–tazobactam.

### 3.2. Antimicrobial Susceptibility Patterns

The antimicrobial resistance profiles of *K. pneumoniae* isolates showed heterogeneous resistance patterns across different classes of antibiotics. All clinical isolates exhibited resistance to ceftriaxone (46/46; 100%). High resistance rates were also observed for ciprofloxacin, piperacillin–tazobactam, azithromycin, gentamicin, and CAZ (44/46; 95.7%). Resistance to carbapenems was observed in 42/46 (91.3%) of isolates (41/46 [89%] to imipenem and 43/46 [93.5%] to meropenem) (Figure 1).

**Figure 1:**
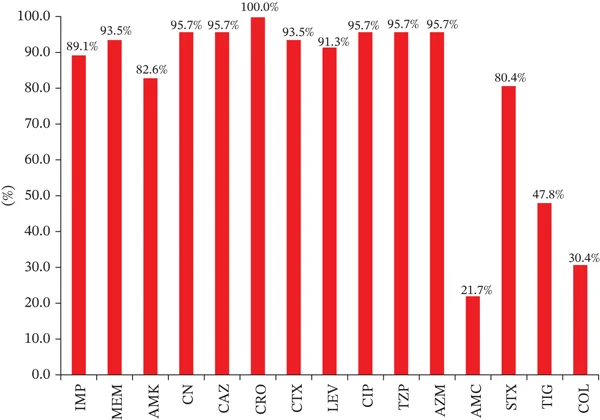
Antimicrobial resistance rates of 46 clinical *K. pneumoniae* strains. IMP, imipenem; MEM, meropenem; AMK, amikacin; CN, gentamicin; CAZ, ceftazidime; CRO, ceftriaxone; CTX, cefotaxime; LEV, levofloxacin; CIP, ciprofloxacin; TZP, piperacillin–tazobactam; AZM, azithromycin; AMC, amoxicillin–clavulanic acid; STX, trimethoprim–sulfamethoxazole; TIG, tigecycline; COL, colistin.

All isolates (10/10; 100%) recovered from the ICU environment were resistant to ceftriaxone, carbapenems, ciprofloxacin, piperacillin–tazobactam, CTX, and azithromycin.

Among the clinical isolates, 36/46 (76%) were confirmed as ESBL producers, and all were identified as MBL producers based on the phenotypic EDTA test. Among the environmental isolates, 8/10 (80%) were classified as ESBL producers, whereas 9/10 (90%) tested positive for MBL production.

Colistin susceptibility was assessed in 46 clinical and 10 environmental isolates using the broth microdilution method. According to CLSI guidelines, 14 clinical isolates (14/46; 30.4%) were resistant, exhibiting minimum inhibitory concentrations (MICs) ≥ 4 *μ*g/mL, whereas one environmental isolate (1/10; 10%) showed resistance with MIC > 4 *μ*g/mL. Details of the clinical and environmental strains are summarized in Tables 2 and 3.

### 3.3. Prevalence of Resistance Genes in Clinical and Environmental Isolates

Carbapenemase genes were detected in all clinical isolates. The most prevalent gene was *bla*_NDM-5_, identified in 45 isolates (45/46; 97.8%), followed by *bla*_OXA-48_ in 26 isolates (26/46; 56.5%) and *bla*_NDM-1_ in one isolate (1/46; 2.2%). Similarly, all environmental isolates carried carbapenemase genes. The *bla*_OXA-48_ gene was detected in all isolates (10/10; 100%), whereas *bla*_NDM-5_ was found in nine isolates (9/10; 90%).

Among the clinical isolates, the most frequent carbapenemase gene combination was the coexistence of *bla*_OXA-48_ + *bla*_NDM-5_, detected in 26 isolates (26/46; 57%). The presence of *bla*_NDM-5_ alone was observed in 19 isolates (19/46; 41%), whereas *bla*_NDM-1_ was identified in a single isolate (1/46; 2.2%). In environmental isolates, the co‐occurrence of *bla*_OXA-48_ + *bla*_NDM-5_ was detected in nine out of 10 isolates (9/10; 90%), whereas *bla*_OXA-48_ alone was found in one isolate from Hospital B (1/10; 10%).

Regarding ESBL genes, *bla*_CTX-M-15_ was the most prevalent among clinical *K. pneumoniae* isolates, detected in 35 isolates (35/46; 76%). The *bla*_SHV_ family was identified in 11 isolates (11/46; 23.9%), including *bla*_SHV-11_ (8/46; 17.4%), *bla*_SHV-12_ (1/46; 2.2%), *bla*_SHV-31_ (1/46; 2.2%), and *bla*_SHV-144_ (1/46; 2.2%). The most common ESBL gene pattern was *bla*_CTX-M-15_ alone in 19 isolates (19/46; 41.3%). The coexistence of *bla*_CTX-M-15_ + *bla*_SHV-11_ was observed in eight isolates (8/46; 17.4%). Less frequent combinations included *bla*_CTX-M-15_ + *bla*_SHV-31_ + *bla*_SHV-12_ in two isolates from Hospital A (2/46; 4.3%), whereas *bla*_SHV-144_ alone was detected in one isolate from Hospital B (1/46; 2.2%). Additionally, *bla*_SHV-11_ alone was identified in a single isolate from Hospital A (1/46; 2.2%) (Tables 2 and 3).

In ICU environmental isolates, ESBL production was detected in 8/10 (80%) of the samples. The *bla*_CTX-M-15_ gene alone was the predominant profile, identified in all ESBL‐producing isolates, whereas the combination *bla*_CTX-M-15_ + *bla*_SHV-11_ was detected in a single isolate from Hospital B. Notably, no *mcr* genes (*mcr*‐1 to *mcr*‐9) were detected in the colistin‐resistant isolates.

### 3.4. Virulence Genes, Biofilm Production, and OmpK36 Typing

Among the 46 clinical *K. pneumoniae* isolates, *rmp*A was detected in 30 isolates (30/46; 65.2%), *iuc*A in 29/46 (63.0%), *peg*1‐344 in17/46 (37.0%), *iut*A in 26/46 (56.5%), and *iro*B in 43/46 (93.5%). The *uge* gene was present in all clinical isolates (46/46; 100%). Regarding the environmental isolates, all of them harbored the *rmp*A, *iro*B, *uge*, and *ybt* genes, whereas lower prevalence was observed for *iuc*A (4/10; 40%), *peg*1‐344 (3/10; 30%), and *iut*A (6/10; 60%).

Thirty‐seven of the 56 isolates (66%) produced positive results in the string test, indicating a hypermucoviscous phenotype. Molecular screening, performed according to Sabença et al. [23], revealed the presence of hvKp‐associated markers with the simultaneous detection of all three genes *iuc*A (aerobactin siderophore biosynthesis), *iut*A (aerobactin receptor), and/or *peg*‐344 (putative transporter) in 10 isolates (10/56; 17.8%). Among 56 *K. pneumoniae* isolates, strong biofilm producers constituted 17/56 (30.36%) of the isolates, followed by moderate producers (10/56; 17.86%), whereas 29/56 (51.80%) of the isolates were classified as nonbiofilm producers.

Based on PCR‐based *OmpK36* typing, Group C was the most prevalent type, accounting for 21/56 (37.5%) of all isolates, followed by Group D (14/56; 25%) and Group B (12/56; 21.4%). Interestingly, among the 17 strong biofilm‐producing isolates, 8/17 (47%) were identified as hvKp belonging to *Omp*K36 Group C and carrying *bla*_NDM-5_ + *bla*_CTX-M-15_ in Libyan patients (Tables 2 and 3).

## 4. Discussion

*K. pneumoniae* is recognized as an opportunistic pathogen frequently linked to ventilator‐associated pneumonia, postoperative infections, and sepsis, accounting for nearly one‐third of Gram‐negative infections in hospital settings. The increasing prevalence of antimicrobial resistance further exacerbates its clinical significance [26]. This study was conducted to assess the detection of virulence genes and to perform genotypic and phenotypic analyses of antimicrobial resistance among cKp and hvKp strains isolated from hospital‐acquired infections in the ICU in Benghazi, Libya.

The distribution of *K. pneumoniae* among different types of hospital‐acquired infections was investigated. Among patients admitted to the ICU, *K. pneumoniae* was most frequently isolated from wound samples (32.6%), followed by blood and urine samples (35%). The *K. pneumoniae* strains isolated from ICU patients exhibited high levels of resistance to several commonly used antibiotics, including ceftriaxone, piperacillin–tazobactam, CTX, CAZ, azithromycin, gentamicin, amikacin, levofloxacin, and ciprofloxacin. This widespread resistance pattern reflects the significant antimicrobial pressure commonly present in the ICU, where the frequent use of broad‐spectrum antibiotics can promote the selection and persistence of MDR strains. The antimicrobial resistance profiles of ICU isolates were largely comparable to those observed among isolates recovered from the hospital environment, suggesting that contaminated surfaces and medical equipment may contribute to the persistence and transmission of these pathogens, facilitating cross‐infection between patients. Similar findings have been reported in several hospital‐based studies where environmental reservoirs played a significant role in maintaining carbapenem‐resistant *K. pneumoniae* within the ICU [27, 28].

Similar resistance trends have been reported in previous studies, which also documented high resistance rates of *K. pneumoniae* to different antibiotics. These findings support the growing concern regarding the dissemination of MDR *K. pneumoniae* in healthcare settings and are consistent with the results of the present study [29, 30].

High resistance to carbapenems was observed among both clinical and environmental *K. pneumoniae* isolates in this study, surmising the possible widespread dissemination of carbapenem‐resistant strains within the hospital setting. Carbapenems are considered last‐line antibiotics for the treatment of infections caused by MDR Gram‐negative bacteria, and resistance to these agents severely limits therapeutic options. Carbapenem resistance in Gram‐negative bacteria is mainly mediated by several mechanisms, including carbapenemase production, activation of efflux pump systems, and decreased outer membrane permeability due to reduced porin expression. Among these mechanisms, the production of plasmid‐mediated carbapenemases represents the most significant contributor to carbapenem resistance [31]. In the present study, all *K. pneumoniae* isolates carried *bla*_NDM_ genes, mainly *bla*_NDM-5_, an emerging and increasingly prevalent variant with potent hydrolytic activity, and *bla*_OXA-48_, a widely disseminated carbapenemase. Interestingly, all the carbapenem‐resistant strains, except one, produce MBL enzymes, phenotypically proven by a positive combined EDTA test and confirmed genotypically by the detection of the *bla*_NDM_ gene. We suggest that the high prevalence of the same carbapenemase‐producing enzyme among all strains could be an alert of the presence of an epidemic outbreak among the healthcare settings in this region. The detection of *bla*_NDM-5_ is particularly alarming due to its recent emergence and frequent association with high‐risk MDR clones [32, 33]. In Tunisia, while *bla*_NDM-1_ has been widely reported, *bla*_NDM-5_ remains rarely documented, highlighting its recent emergence [34]. Furthermore, no *bla*_KPC_ or *bla*_VIM_ genes were identified in our isolates. However, previous research conducted in Libya reported the presence of *bla*_NDM-1_, *bla*_VIM-1_, and *bla*_OXA-48_ in *K. pneumoniae* isolates recovered from both patients and healthcare workers, highlighting regional variability in the distribution of carbapenemase genes [28]. These differences may reflect variations in antibiotic usage, infection control practices, and local epidemiology of resistant clones across healthcare settings.

In this study, the carbapenemase genes *bla*_NDM_ and *bla*_OXA-48_ were detected in *K. pneumoniae* isolates in association with ESBL genes, particularly *bla*_CTX-M-15_ and *bla*_SHV_. This association was observed in our study in both clinical and environmental isolates. Similar patterns of coexistence involving *bla*_NDM_, *bla*_OXA-48_, and *bla*_VIM_, together with ESBL genes, have often been described in clinical isolates from several countries, including Tunisia, Palestine, and Egypt [34–36]. The simultaneous presence of carbapenemase and ESBL determinants in the same strain highlights the accumulation of multiple clinically relevant resistance mechanisms. Such genetic combinations are particularly alarming, as they can significantly limit therapeutic options and promote the emergence and dissemination of MDR *K. pneumoniae* in healthcare settings. Consequently, infections caused by these strains are often associated with therapeutic failure, prolonged hospitalization, and increased morbidity and mortality.

The prevalence of ESBL production among members of the *Enterobacteriaceae*, particularly *Escherichia coli* and *K. pneumoniae*, is among the highest reported worldwide [37, 38]. Among ESBL‐encoding genes, the CTX‐M family has become the most predominant, with the CTX‐M‐15 variant being the most widely disseminated globally and representing a major concern in healthcare settings due to its rapid spread and strong association with MDR strains [38]. Nevertheless, the detection of *bla*_SHV_ and *bla*_TEM_ genes does not always correlate with phenotypic ESBL production, since only specific variants of these genes confer an ESBL phenotype. Among our strains, *bla*_CTX-M_ genes were detected in 76% of *K. pneumoniae* isolates recovered from patients and 80% from the ICU environment. These findings are consistent with other studies, which also reported a high prevalence of *bla*_CTX-M_ genes among clinical *K. pneumoniae* isolates [27, 38]. In contrast, the *bla*_SHV_ gene was identified in 19.6% of clinical isolates and in 20% of environmental ICU isolates. The detected variants included *bla*_SHV-11_, *bla*_SHV-12_, *bla*_SHV-31_, and *bla*_SHV-144_. Notably, to the best of our knowledge, these *bla*_SHV_ variants have not previously been reported in *K. pneumoniae* isolates from Libya, suggesting a possible emergence or underreporting of these ESBL determinants in the region.

In this study, ESBL‐associated SHV variants, including SHV‐12 and SHV‐31, were detected among the *K. pneumoniae* isolates. The presence of diverse SHV‐type ESBL variants in *K. pneumoniae* has been reported in several regions. For instance, SHV‐5, SHV‐12, and SHV‐33 were identified in isolates from Palestine [39], whereas studies from Saudi Arabia reported the presence of SHV‐5 and SHV‐12 [40]. In this study, two *K. pneumoniae* isolates coharbored two ESBL genes, *bla*_CTX-M-15_, together with either *bla*_SHV-12_ or *bla*_SHV-131_. Similar findings have been previously reported, where the coexistence of CTX‐M‐15 with SHV‐12 or SHV‐5 was documented in *K. pneumoniae* isolates [40–42]. In addition, four clinical isolates carried the *bla*_CTX-M-15_ gene despite yielding negative results in the double‐disk synergy test, suggesting a discrepancy between genotypic and phenotypic detection of ESBLs. Such false‐negative phenotypic results may occur due to low gene expression, methodological limitations, or the presence of additional *β*‐lactamases, such as AmpC enzymes or carbapenemases masking the clavulanate synergy effect [6].

In this study, a significant proportion of *K. pneumoniae* isolates (*n* = 15, 32.6%) exhibited resistance to colistin. These results align with previous reports from Libya (2015–2018) [43, 44], highlighting a persistent presence of colistin‐resistant *K. pneumoniae* in both clinical and hospital environmental settings. The detection of such highly resistant strains is particularly concerning, as colistin remains a last‐resort antibiotic for MDR Gram‐negative infections. Their continued circulation in the ICU environment underscores the urgent need for enhanced infection control measures and rigorous antimicrobial stewardship to prevent further dissemination. Despite phenotypic colistin resistance, none of the isolates carried *mcr*‐1 to *mcr*‐9 genes, indicating that resistance is likely mediated by chromosomal mechanisms, such as mutations in *mgr*B or *pmr*A/*pmr*B [45].

Among the investigated virulence genes, the *uge* gene was detected in all isolates, suggesting that it represents a conserved virulence determinant within the studied population. This gene has been widely associated with lipopolysaccharide biosynthesis and contributes to bacterial fitness and pathogenicity. In addition, the *ybt* and *iro*B genes were detected at relatively high frequencies, indicating that iron acquisition systems are common among these isolates and may contribute to their virulence potential. The presence of *ybt*, which encodes the yersiniabactin siderophore system, is particularly noteworthy because it enhances bacterial virulence by promoting iron acquisition and facilitating biofilm formation, thereby improving bacterial survival and persistence within host environments. Similarly, *iro*B, a component of the salmochelin siderophore gene cluster, plays a critical role in iron uptake, a key factor for bacterial growth and successful host colonization [46]. The detection frequencies of *Wab*G, *uge*, *iuc*A, *iut*A, and *ybt* genes are consistent with those recently reported in Russia and China [29, 47]. However, variation was noted in the distribution of the other virulence‐associated genes. These differences may reflect geographic heterogeneity, diversity in circulating *K. pneumoniae* lineages, or selective pressures driven by local clinical practices and environmental conditions.

Phenotypically, 66% of the isolates exhibited a positive string test, and all these strains carried the *uge*, *WabG*, *ybt*, and *iroB* genes, suggesting the presence of a hypermucoviscous phenotype. Nevertheless, increasing evidence indicates that the string test alone lacks sufficient sensitivity and specificity for the accurate identification of hvKp [8, 48]. Therefore, reliance on this phenotypic assay may lead to misclassification of hvKp strains, emphasizing the importance of combining phenotypic and molecular approaches for reliable detection.

Among clinical isolates, 30 strains (65%) carried more than three virulence biomarkers, including *rmp*A, *iucA*, *peg*1‐344, *iut*A, and *iro*B, whereas only two isolates (6.7%) possessed four of the key markers (*iucA*, *iroB*, *peg-344*, and *rmpA*). In contrast, the *peg*2‐344 and *fim*H genes were not detected in any of the clinical and environmental isolates. Although both variants, *peg*1/2‐344, are associated with the hypermucoviscous phenotype and virulence potential, differences in their distribution have been reported among hvKp lineages, and the absence of *peg*2‐344 may reflect the limited circulation of strains harboring this variant. In contrast, the *peg*2‐344 and *fim*H genes were not detected in any of the clinical and environmental isolates. These findings highlight the genetic diversity of virulence profiles among *K. pneumoniae* isolates and support the need for comprehensive molecular characterization to better understand the emergence of potentially hypervirulent and MDR clones.

Interestingly, all isolates recovered from the ICU environment consistently harbored the *uge*, *rmp*A, *Wab*G, *ybt*, and *iro*B genes, suggesting a higher virulence potential compared with patient‐derived isolates. This finding highlights the hospital environment, particularly ICU settings, as a potential reservoir of highly virulent *K. pneumoniae* strains that may facilitate the persistence and dissemination of nosocomial infections. Three genes linked to the hypervirulent phenotype, as defined by Russo et al. [8], were detected in 10 isolates. However, using the more stringent criteria proposed by Russo et al. [48], which require the simultaneous presence of five key biomarkers (*iuc*A, *iro*B, *peg-*344, *rmp*A, and *rmp*A2) for reliable identification of hvKp, the number of potentially hypervirulent isolates in our collection would likely be lower. These results highlight the need for standardized molecular criteria to enhance surveillance, risk assessment, and epidemiological monitoring of hvKp. Previous studies have demonstrated that the rapid dissemination of conjugative *bla*_NDM-_bearing plasmids plays a critical role in the emergence and global spread of CR‐hvKp, posing an increasing threat to public health [49, 50]. To our knowledge, this study is the first report from Libya characterizing the virulence gene profiles of *K. pneumoniae*.

The *K. pneumoniae* species can form biofilms, largely mediated by structural components such as the capsule and pili [51]. These biofilms act as protective barriers that reduce antibiotic susceptibility, contributing to decreased sensitivity to agents such as gentamicin, ampicillin, and ciprofloxacin [52, 53]. Phenotypic assessment of biofilm formation revealed that 11 isolates (19.6%) exhibited strong biofilm‐forming capacity, whereas 15 isolates (26.8%) demonstrated a moderate biofilm phenotype. Molecular analysis showed that all strong biofilm‐producing isolates consistently carried the *uge*, *ybt*, *iro*B, and *Wab*G genes, whereas most also harbored *rmp*A, *mrk*D, and *iuc*A. In contrast, isolates with moderate biofilm‐forming ability uniformly possessed *uge*, *ybt*, *iroB*, and *WabG* but exhibited a lower prevalence of *rmp*A, *mrk*D, and *iuc*A. These findings suggest that while some virulence genes are widely distributed among isolates, additional determinants may enhance the capacity for robust biofilm production. Interestingly, approximately 40% of isolates recovered from the ICU environment exhibited strong biofilm‐forming ability; however, most of these strains lacked the *iuc*A and *mrk*D genes. Otherwise, a recent study reported that the *mrk*D and *Wab*G virulence genes do not have a significant relationship with biofilm formation. This same study showed a highly significant association between the *fim*H and the *wca*G virulence genes and biofilm formation [54]. Overall, the widespread distribution of these virulence‐associated genes may provide a selective advantage, facilitating bacterial persistence and pathogenicity in both clinical and environmental settings.

Furthermore, PCR‐based *Omp*K36 typing revealed that Group C was the most prevalent genotype in our collection, accounting for 37.5% of the isolates. This distribution is comparable to findings reported in several Asian countries, particularly in Iran and Taiwan [23, 25]. However, porin‐based typing methods such as OmpK36 classification have certain limitations. In the present study, nine *K. pneumoniae* clinical isolates could not be typed, which may be related to the high genetic diversity of outer membrane porin genes in *K. pneumoniae* populations [55]. Moreover, this approach does not reliably reflect recent horizontal gene transfer events or the acquisition of plasmid‐mediated resistance determinants. Alterations in OmpK36 expression or structure, resulting from mutations or regulatory changes, may also affect antimicrobial susceptibility independently of sequence type or porin group. Therefore, although OmpK36 typing provides a rapid and cost‐effective tool for assessing isolate relatedness, its results should be interpreted with caution and ideally complemented by higher resolution molecular methods, such as multilocus sequence typing or whole‐genome sequencing.

Our findings provide an overview of the characteristics of carbapenem‐resistant *K. pneumoniae* isolates from two Libyan hospitals, with particular emphasis on antimicrobial resistance profiles and the distribution of virulence determinants among the studied strains. However, this study included only 56 carbapenem‐resistant isolates collected from two healthcare facilities over a 6‐month period. The relatively small sample size represents an inherent limitation that may restrict the generalizability of our findings. Therefore, larger multicenter studies covering different geographic regions and longer surveillance periods are warranted to provide a more comprehensive understanding of the epidemiology, resistance mechanisms, and virulence profiles of carbapenem‐resistant *K. pneumoniae* in Libya.

## 5. Conclusion

This study reveals the widespread presence of MDR and potentially hvKp strains among both ICU patients and the hospital environment and therefore provides valuable baseline data on the epidemiology of MDR and hvKp in Libya, where information on this pathogen remains scarce. The high prevalence of carbapenemase genes, together with the frequent detection of virulence determinants and biofilm‐forming capacity, highlights the growing threat posed by these pathogens in healthcare settings. These findings emphasize the urgent need for strengthened infection control strategies, continuous epidemiological surveillance, and rational antibiotic use to limit the spread of these highly adaptable and clinically significant bacteria.

## Funding

No funding was received for this manuscript.

## Ethics Statement

This study was conducted in accordance with the ethical standards of the relevant institutional and national research committees. Ethical approval was obtained from the Medical Research Ethics Committee of Benghazi Central Hospital and Al‐Jalaa Hospital, Benghazi, Libya. Bacterial strains were analyzed anonymously, and environmental samples were collected as part of routine infection control surveillance.

## Conflicts of Interest

The authors declare no conflicts of interest.

## Data Availability

The data that support the findings of this study are available from the corresponding author upon reasonable request.
